# Supplementary material for: ENSO impacts child undernutrition in the global tropics
Source: Nat Commun. 2021 Oct 12;12:5785. doi: 10.1038/s41467-021-26048-7 (PMC8511020; doi:10.1038/s41467-021-26048-7)
Supplement: Supplementary file 1 — Supplementary Information [file 41467_2021_26048_MOESM1_ESM.docx]

**Supplementary Information for**

ENSO Impacts Child Undernutrition in the Global Tropics

Jesse K. Anttila-Hughes, Amir S. Jina, Gordon C. McCord

**This file includes:**

Supplementary Information Text

Supplementary Figures 1 to 4

Supplementary Tables 1 to 15

Supplementary References 1 to 4

Supplementary Information Text

**Robustness of empirical results.** The association between the ENSO index and undernutrition remains robust to a variety of model specifications controlling for different plausible observable and unobservable factors. Supplementary Table 1 shows the build-up of our basic model for weight-for-age and underweight to illustrate this point. Panel A column (1) replicates the results shown in Table 1 of the paper, to aid comparison to other columns. Model (2) flexibly detrends the data by (tropical) year of interview, and normalizes by interview month and country using fixed effects to provide a plausibly causal estimate that is not confounded by trends, seasonality and country of survey. The association between NINO3.4 and weight-for-age is statistically significant at 95% confidence levels, with opposite signs in areas that have a positive correlation between precipitation and NINO3.4 and those that do not. Note that the coefficient of -0.026 for places without positive correlation (93.6% of observations) is statistically consistent for all specifications. Model (3) normalizes data by rural/urban location and adds child- and mother-level controls (mother’s age at child’s birth, total years of mother’s education), resulting in a decrease in the coefficient for positive precipitation correlation locations but no change for other locations. Model (4) flexibly detrends the data separately by UNICEF region^[[1]](#footnote-2)^ and adds country-specific rural/urban fixed effects, resulting in no statistical change in the coefficients while increasing significance levels to 99% confidence. Model (5) adds country-specific interview month fixed effects to remove seasonality separately by country, rather than by the larger UNICEF region. Because the DHS are collected with idiosyncratic timing within the year, there are often months within a country that are only observed once. This means that a country-by-month fixed effect would be collinear with ENSO state for children measured in those singly-observed months, since all children observed within that month get the same annual ENSO value, meaning they do not contribute any identifying variation in ENSO to the main regression. Regardless, while this stricter specification makes estimates slightly noisier, they are not statistically different from those in (4). Model (6) controls for mother and child characteristics separately by country, resulting in no significant change in results. Finally, model (7) replaces the country fixed effects with fixed effects in first level administrative unit, thus comparing children living in the same state/province interviewed during different ENSO states. This stricter specification also does not lead to changes in results. Panel B performs all the same specifications using the binary outcome of underweight (below -2σ in weight for age). The pattern across specifications is similar: effects are consistent in direction and magnitude across all models, with statistical significance emerging after normalizing data by rural/urban location and adding child- and mother-level controls in model (3).

Supplementary Table 2 presents models that vary observation weights to inform interpretation. Models (1)-(4) show results on weight for age, while (5)-(8) show results on underweight (below -2σ in weight for age). Models (1) and (5) use weights as in main results in Table 1 in order produce estimates interpreted as ENSO’s effect on a child in the average country. These weights use the DHS sampling weights, adjust for survey size differences across countries, and then adjust for different numbers of DHS surveys in different countries:

$\omega_{\mathrm{isc}}^{\mathrm{avgcountry}}=\left( \frac{\mathrm{DHSweigh}t_{\mathrm{isc}}}{\mathrm{surveysiz}e_{\mathrm{sc}}} \right)\left( \frac{1}{\mathrm{totalsurvey}s_{c}} \right)$ (4)

Models (2) and (6) weight observations such that results are interpreted as the effect on the average child in the sample countries. The weights are the same as above, but now weight across countries by country population:

$\omega_{\mathrm{isc}}^{\mathrm{avgchild}}=\left( \frac{\mathrm{DHSweigh}t_{\mathrm{isc}}}{\mathrm{surveysiz}e_{\mathrm{sc}}} \right)\left( \frac{\mathrm{populatio}n_{c}}{\mathrm{totalsurvey}s_{c}} \right)$ (5)

Results with these weights suggest larger effects of ENSO, although estimates are less precisely identified in the case of weight for age (2). Given that India represents 23% of the data and is by far the country with the largest population, models (3) and (7) use average child weights but exclude India and find that results on the average child are larger than in (1) and (5) but not statistically different. In order to gauge whether the results might be driven by observation weights, models (4) and (8) use no weights and find results very similar to the main model. We maintain that weights are appropriate for the main specification given the stratified nature of DHS sampling and the fact that countries have different numbers of DHS surveys.

Supplementary Table 3 columns (1)-(5) show alternative definitions of the ENSO variable for models on weight for age, while columns (6)-(10) are on underweight (below -2σ in weight for age). While our main model uses the mean monthly value of the May-Dec anomaly, models (1) and (6) use the maximum monthly value observed over the full (May-April) tropical year. Models (2) and (7) use the maximum over the May-Dec tropical year; models (3) and (8) use the mean over the tropical year; and models (4) and (9) use the maximum of a three month rolling mean of monthly values observed during the full tropical year. Columns (5) and (10) use indicator variables for El Niño-like and La Niña-like states designating all years where the maximum of a three month rolling mean of monthly values was greater than 0.5°C (Niño-like) or less than -0.5°C (Niña-like) from its 1981-2010 climatology, following the NOAA threshold. We note that in every case the association between decreased child weight for age and increased ENSO values remains, with some variation. The binary indicator for El Niño-like events is associated with lower weight for age in (5) and higher risk of underweight in (10), but is not statistically significant except for the correspondingly opposite effects in regions with positive precipitation correlation to ENSO. The same patterns hold for the La Niña indicator, with opposite signs to the El Niño indicator throughout.

Supplementary Table 4 estimates effects of ENSO on the same anthropometric measures as Table 1, but allowing for different effects by child age categories of 0-5 months, 6-11 months, 12-23 months, 24-35 months and 36-59 months. With few exceptions, coefficients are consistent in sign and magnitude across age groups for each outcome variable and with the corresponding coefficient in Table 1. The significantly smaller sample size in each age category results in imprecise estimates, however. The 36-59 months category has a larger sample and correspondingly higher precision in the estimates.

Supplementary Table 5 does not distinguish effects by the precipitation correlation to NINO3.4 to produce an average treatment effect of warmer ENSO across all children. The average effect is consistent with the effect size in Table 1 on children outside areas where precipitation is positively correlated to NINO3.4. The average effect is that warmer ENSO leads to a 0.04σ/°C reduction in weight-for-age (p=0.02), and a 1 percentage point increase in prevalence of underweight (p<0.01).

Supplementary Table 6 implements an alternative detrending of the data using decade fixed effects and shows that coefficients are not statistically different from Table 1. Supplementary Table 7 explores the lagged effects of ENSO by adding a one-year lag to the specification. There is only evidence of persistent effects of warmer ENSO on child nutrition in the subsample with positively correlated rainfall. Supplementary Table 8 shows evidence for the persistent effects of ENSO by focusing on older children (ages 2-5 years) and the effect of the ENSO state during year of birth and during two subsequent years^1^ on height for age and likelihood of being stunted. While weight can recover quickly after a negative shock, child height is a slower-moving, cumulative anthropometric indicator and shows the longer-lasting effects of ENSO state on child nutrition during early life.

Supplementary Tables 9-10 test whether results are robust to alternative definitions of teleconnection. Figure 1C and 1D shows the global map of ENSO correlations to precipitation and temperature used for teleconnection assignment. Supplementary Table 9 extends the main sample to include countries that are teleconnected with NINO3.4 in terms of precipitation but not temperature. Specifically, we added countries that are not already in the main sample, have DHS surveys and have at least 30% of their land area showing correlation between precipitation and NINO3.4. These countries are Kyrgyz Republic, Azerbaijan, Armenia, Turkey, Pakistan, Kazakhstan, and Egypt. No coefficient is statistically different from its analogue in Table 1, and the coefficients for WAZ, BMI and wasted for the positive precipitation correlation sample are now statistically significant. Supplementary Table 10 restricts the sample to include only those countries which have both a significant teleconnection with ENSO via temperatures and precipitation. This leads to a strictly smaller subsample of countries than the main estimates in Table 1. The sample size drops to approximately 250,000, a substantial change compared to approximately 1.25 million for the main sample, and 1.35 million for the extended sample in Supplementary Table 9. We note that there is a priori ambiguity on whether the effects of ENSO in this smaller sample would be larger or smaller. On the one hand, the subsample represented by this table are those with presumably stronger (or at least more noticeable) climate variability induced by ENSO. This could imply that these countries are better adapted, and hence might display smaller effects of ENSO on outcomes. On the other hand, a number of other results would imply that, as a set of lower income countries, their ability to adapt to this climate variability may be limited. Therefore, by restricting to countries with stronger ENSO effects, we might expect that the effects would be stronger. The results in the table are supportive of the second hypothesis, i.e., worsening effects, but we they are nonetheless not statistically different from the results in Table 1.

Supplementary Figure 1 explores the robustness of our teleconnection calculation both across datasets and across NINO indices. Specifically, it shows the teleconnections as measured by the number of months of statistically significant correlations at a pixel level. A pixel is teleconnected if its temperature is closely coupled to ENSO, defined as having local temperature in month *t* significantly correlated with the second month lag (*t – 2*) of the ENSO state for at least three months of the year. Differences over land using the UDEL dataset (which does not contain temperatures over the oceans) with NINO3, NINO3.4, and NINO4 were minimal. Supplementary Figure 1 shows the number of teleconnected months over land and oceans using the ECMWF ERA-Interim reanalysis for NINO3, NINO3.4, and NINO4 indices. The pattern over land remains largely unchanged, and the spatial distribution over land for the NINO3.4 plot produces an extremely similar set of teleconnected countries as the UDEL-teleconnections used in the main analysis.

Supplementary Figures 2-3 and Supplementary Tables 12-13 explore whether the ENSO state might affect the timing of DHS surveys within the year, and therefore spuriously lead to changes in child anthropometrics due to seasonality. Supplementary Figure 2 plots sample sizes by month and ENSO state with 95% CIs to show variation in these patterns across DHS surveys. There is no statistically different monthly pattern across ENSO states. Supplementary Figure 3 confirms that the effect of ENSO on weight for age is consistent regardless of the season during which the child was surveyed. Supplementary Table 12 adds country-specific monthly fixed effects to ensure that only children interviewed in the same country during the same month of the year are being compared across ENSO states in their anthropometrics. Effects of ENSO are very similar to Table 1 in the case of weight for height and BMI, and a bit smaller in magnitude in the case of WAZ and wasting, although the coefficients are not statistically distinguishable from those in Table 1. Supplementary Table 13 regresses the DHS month of interview on the ENSO state (including all covariates, detrending and fixed effects in the main regression) and shows that the two are not associated. These results suggest that the effects of ENSO state on child anthropometrics are not the result of correlated interview timing changes.

Supplementary Table 14 employs a variety of standard error clustering approaches in order to adjust for a covariate shock (all observations experience the same ENSO treatment variable per year) as well as spatial and serial correlation in child anthropometric estimates. Inference remains consistent whether standard errors are not clustered (1); clustered at UNICEF region (2), country (3) or admin1/state (4) to adjust for arbitrary serial and spatial autocorrelation at these geographic units. Inference is also consistent under two-way clustering^2^ at UNICEF region & decade (5), country and year (6), or admin1 and year (7, the main specification). Note that (7) is designed to address possible spatial and temporal autocorrelation through use of two-way clustering of standard errors^32^ at the levels of interview year and first subnational administrative subunit (e.g., state or province, at which level the DHS is representative). This approach is conservative, and controls for arbitrary autocorrelation within both the level of treatment (i.e. tropical year) across space as well as within the DHS sample frame across multiple surveys at different times.

Supplementary Table 15 implements a logistic version of regressions 4-5 in Table 1 which have dichotomous dependent variables. Columns (1) and (3) are the results in Table 1 of the paper using the linear probability model for the underweight and wasted binary outcomes. Columns (2) and (4) show results for the corresponding logit specifications, reporting odds ratios. The results are qualitatively unchanged from the LPM results, with a positive ENSO anomaly generating statistically significant higher odds of being underweight, and lower odds in areas with positive precipitation correlation to warmer ENSO.

Finally, Supplementary Figure 4 shows that a randomization inference test rejects the possibility that the structure of the data is spuriously resulting in the estimated effects on child anthropometrics^3^. The procedure randomly permutes yearly NINO3.4 values across years, and estimates the main specification on child weight for age (as in Table 1 column 1, excluding the observations with positive precipitation correlation to NINO3.4):

$Y_{ict}=\alpha+\beta_{n}\mathrm{NINO}_{t}+\gamma\cdot\mathbf{X}_{\mathrm{ic}}+f\left( t_{\mathrm{UNICEF}} \right)+FE_{\mathrm{cr}}+\varepsilon_{ict}$ (6)

The figure plots the distribution of $\beta_{n}$ estimated from the 2,000 random permutations of NINO3.4. This, in effect, creates 2,000 placebo datasets where every child surveyed in tropical year T, i.e., between May of calendar year T and April of calendar year T+1, is assigned a random NINO3.4 anomaly value from the time series of NINO3.4 without replacement. The distribution of estimates from running our main specification on these 2,000 datasets is the blue shaded region. It is centered at 0, providing evidence that this research design is not biased by, for example, temporal autocorrelation. Further, the coefficient we estimate using the actual NINO3.4 variation (-0.025) is substantially different from zero (p = 0.08). This Fisher randomization inference test illustrates that the residual annual variation in NINO3.4 and the child anthropometric data is meaningful compared to arbitrary degrees of freedom at the annual level.

**Calculations for Figure 3.** The coefficient on the average treatment effect regression (without splitting the sample by the sign of the precipitation correlation) is -0.04 (see Supplementary Table 5). The effect of the 1.92°C increase in SST during the 2015 El Niño is therefore 1.92*-0.04 = -0.078 in z-score units applied to a total of 310,833,752 children ages 0-4 in the sample countries in 2015 (data from the World Bank’s World Development Indicators). Since the standard deviation of the weight-for-age z-scores in our sample is 1.51, then the effect size becomes -0.078/1.51 or -0.052, which multiplied by the number of children yields an aggregate change of standardized z-score units of -16,023,557.

In the case of provision of complementary foods, Bhutta et al. (2013)^4^ review 16 trials and quasi-experimental studies and document significant effects on weight-for-age of 0.26 (0.04-0.48) in standardized mean difference. This leads to an estimate of 62 million (16 million / 0.26) children requiring complementary food interventions to offset the adverse effects of the 2015 El Niño. A similar calculation is done to produce the confidence interval (we use the higher value of 0.48 in effect size CI to provide more conservative estimates on the number of children, corresponding to 33 (16/0.48) million children, and provide a CI of 33-90 million children).

In the case of nutrition education, the Bhutta et al. (2013) review suggests significant effects on weight-for-age z-scores of 0.26 (0.12-0.41) in standardized mean differences. This leads to an estimate of 62 million (16 million / 0.26) food insecure children receiving nutrition education to offset the adverse effects of the 2015 El Niño. A similar calculation is done to produce the confidence interval (we use the higher value of 0.41 in effect size CI, corresponding to 39 (16/0.41) million children, and provide a CI of 39-84 million children).

Finally, in the case of multiple micronutrient supplementation, Bhutta et al. (2013) systematically review 18 trials mostly in developing countries and find an effect size of 0.14, suggesting that around 114 million children (16 million / 0.14) would need to receive multiple micronutrient supplementation to offset the 2015 El Niño. A similar calculation is done to produce the confidence intervals. The effect size CI of multiple micronutrient supplementation is 0.03-0.25. We calculate symmetric CIs for the number of children requiring the public health intervention using the high CI on the intervention effect size, which generates a more conservative (smaller) calculation on the effect of El Niño. In this case, the higher CI value of 0.25 corresponds to 64 million children (18.8/0.25), and we apply the same CI range on the higher number of children to arrive at a CI of 64-165 million children.


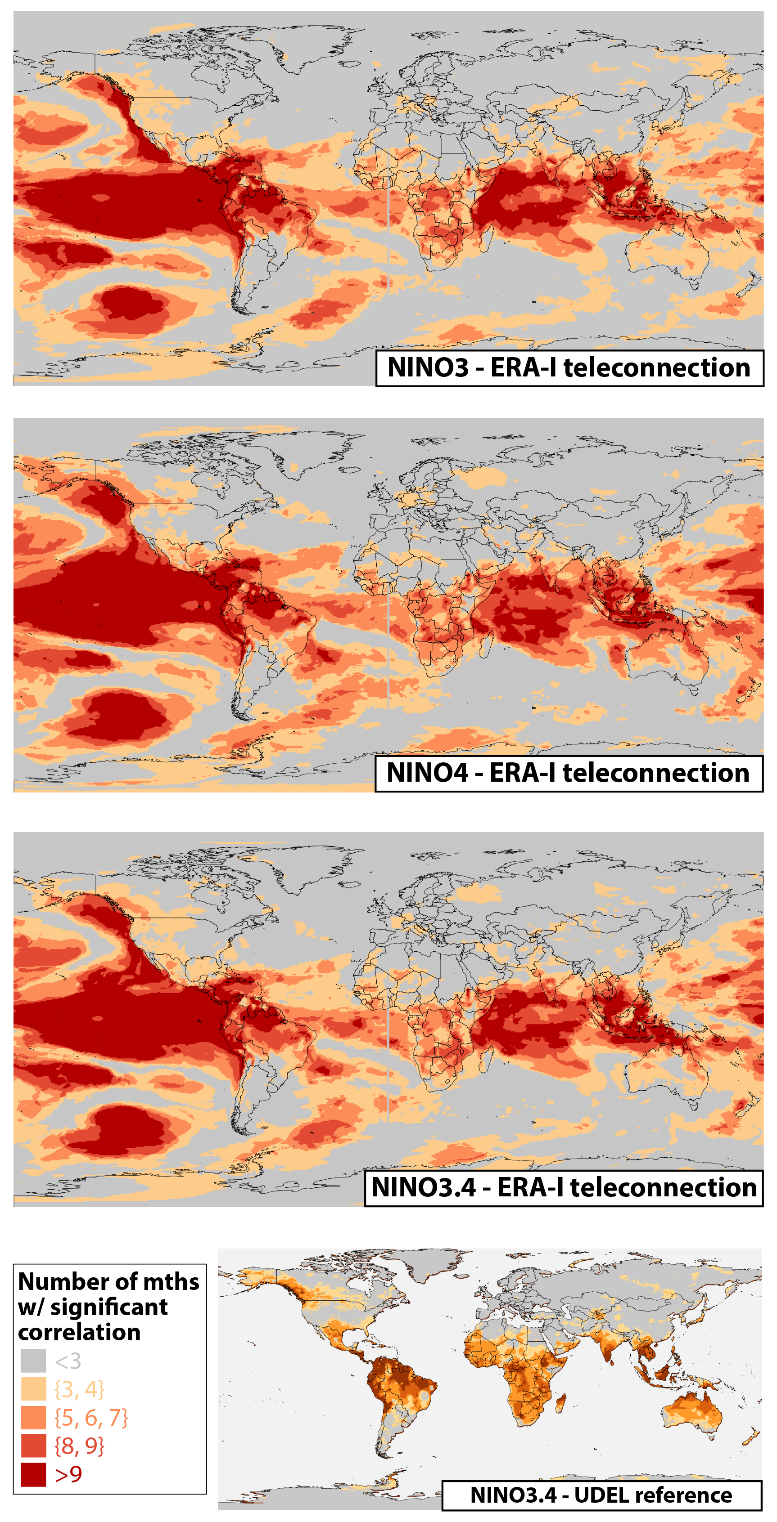


Supplementary Figure 1. Pixel-level monthly correlation of surface temperature (1980-2010) and two-month lag of NINO3.4 Sea Surface Temperature (SST), showing teleconnections. Values represent number of months in a year significantly associated with ENSO at the 10% level. The top three panel show teleconnections calculated using three different NINO indices. The bottom panel gives a comparison to the values over land for NINO3.4 from the climate dataset employed in the main analysis.


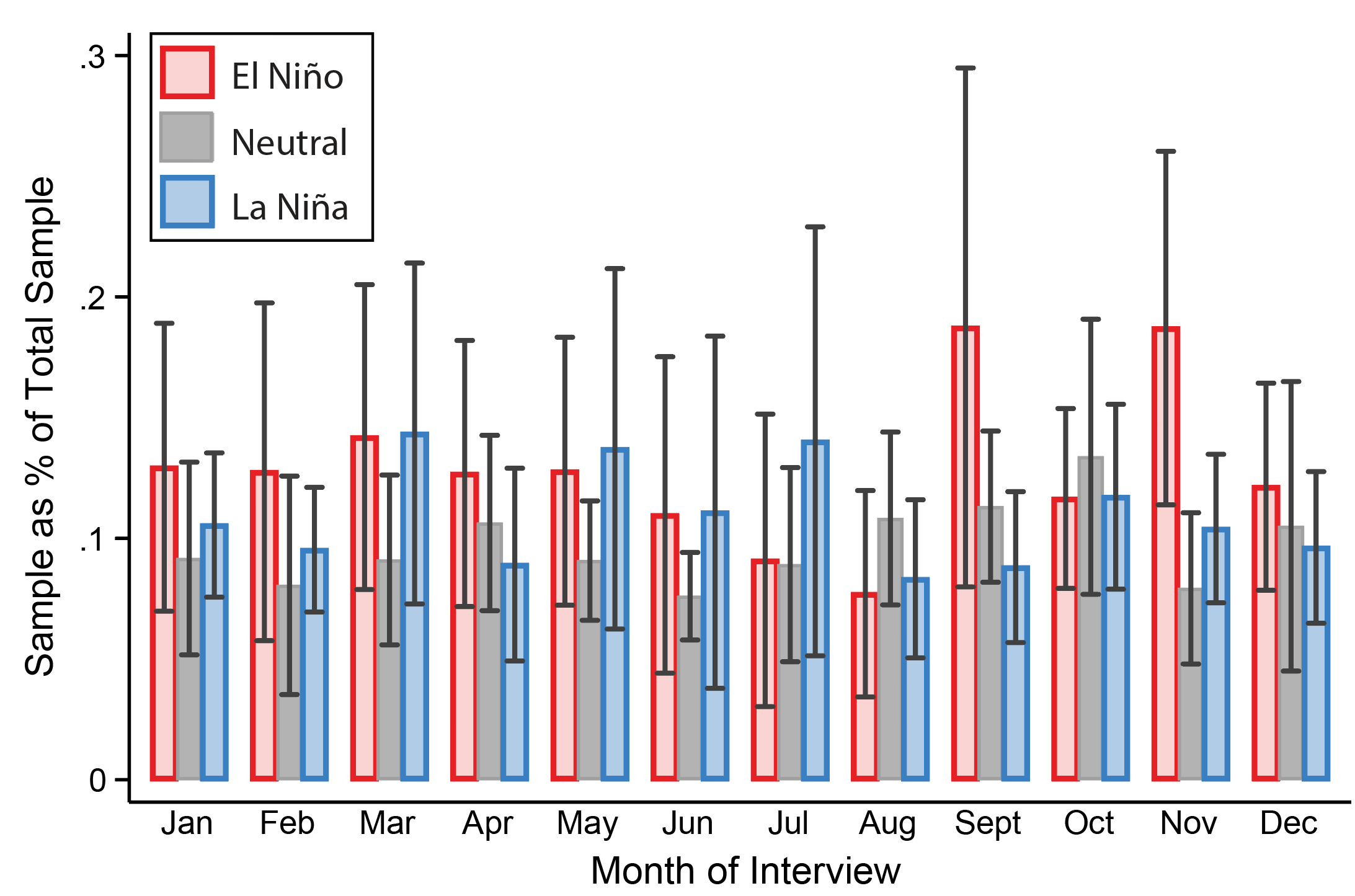


Supplementary Figure 2. Sample sizes show no statistically different monthly pattern in DHS interviews across ENSO states. Graph plots the result of calculating the sample percentage of each DHS survey (n= 1,253,176 children across 51 surveys) separately by month and ENSO state. Bar heights and error bars indicate mean and 95% confidence interval across DHS surveys.


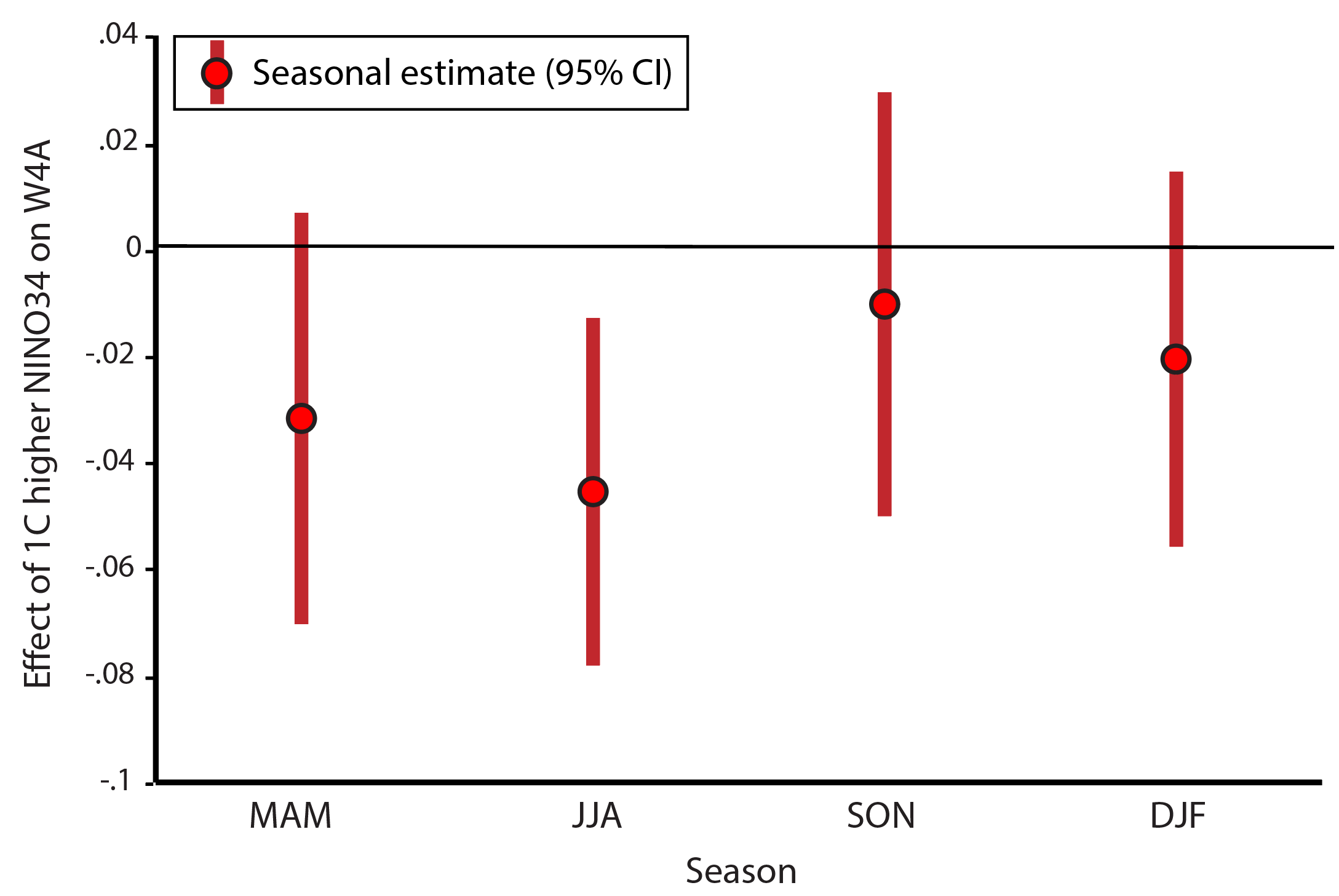


**Supplementary Figure 3. ENSO effects on weight for age are consistent across seasons of DHS survey.** Coefficients on regression of weight-for-age (n= 1,253,176 children across 51 surveys) on ENSO effects measured in °C, allowing for different effects by season of the tropical year (March-April-May, June-July-August, September-October-November, December-January-February). Estimates are from OLS regressions with controls consisting of: fixed effects (indicators) for each country; country-specific mother's age at child's birth, total years of mother's education, and rural vs. urban indicator; and UNICEF world region-specific linear trends in survey year and fixed effects for month of interview. Standard errors are two-way clustered at the level of tropical year and subnational administrative unit, and observations are reweighted using DHS sample weights and country size weights in order for estimates to be representative for an average country. Asterisks indicate statistical significance at the 1% (***), 5% (**) and 10% (*) levels (two-sided t-test, single hypothesis).


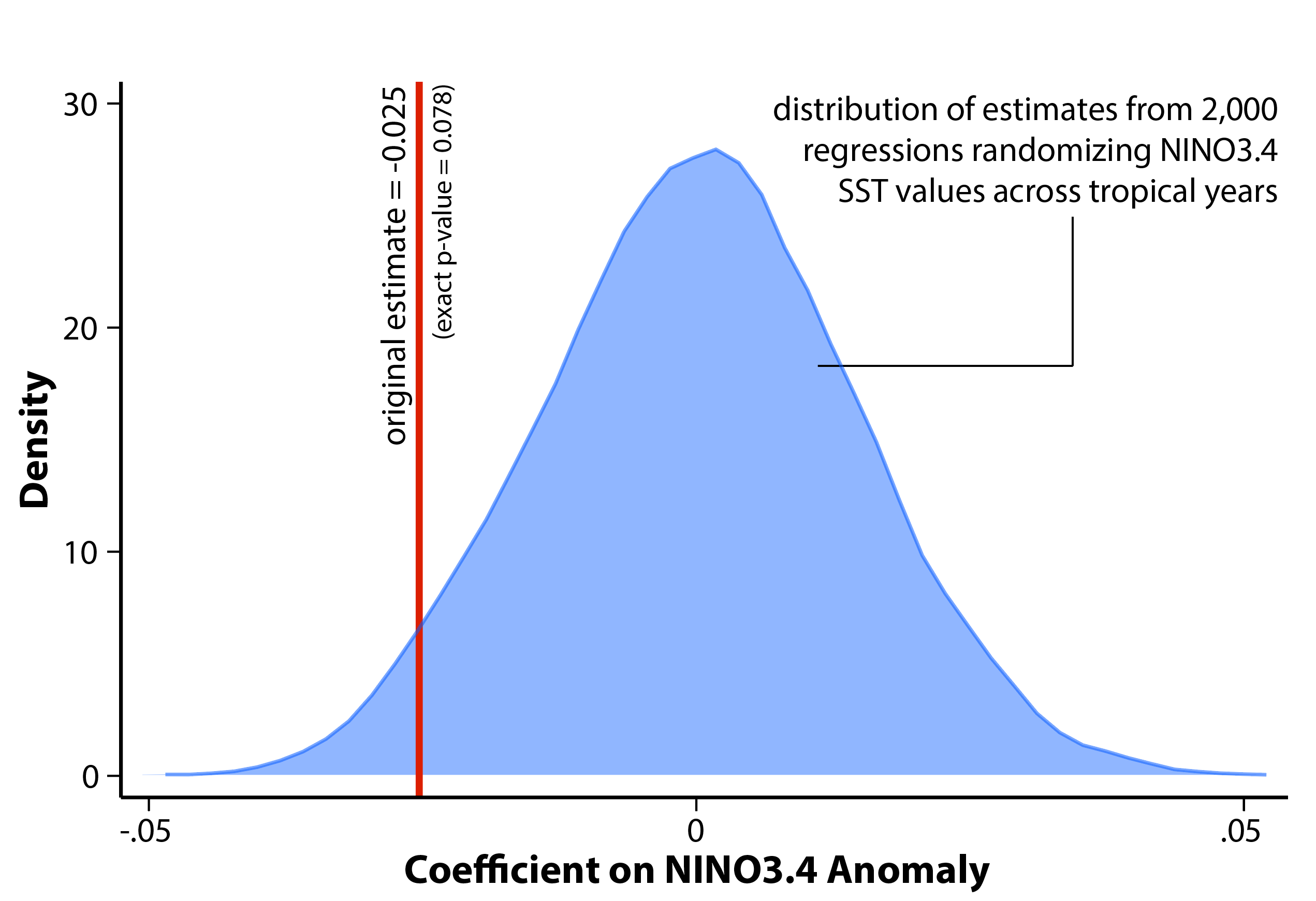


**Supplementary Figure** **4. Randomization inference test indicates association of ENSO state and nutrition unlikely due to chance.** The figure plots the distribution of coefficients 𝛽_n_ from the following regression matching the estimation in column 1 of Table 1 after excluding locations with positive precipitation correlation:  $Y_{ict}=\alpha+\beta_{n}\mathrm{NINO}_{t}+\gamma\cdot\mathbf{X}_{\mathrm{ic}}+f\left( t_{\mathrm{UNICEF}} \right)+FE_{\mathrm{cr}}+\varepsilon_{ict}$*. Y* is child weight for age.  Estimates of $\beta_{n}$ are produced after values of NINO3.4 are randomly reassigned across tropical years.  The process is repeated with 2,000 random permutations of NINO3.4. The randomized permutation exercise rejects the possibility that the estimated $\beta_{n}$ of -0.025 (shown by the vertical line) is a spurious result (p = 0.08, two-sided t-test, single hypothesis).  Estimates are from OLS regressions with controls consisting of: fixed effects (indicators) for each country; country-specific controls for mother's age at child's birth and total years of mother's education; country-specific fixed effects for rural vs. urban; and UNICEF world region-specific linear trends in survey year as well as fixed effects for month of interview. Observations are reweighted using DHS sample weights and country size weights in order for estimates to represent an effect on the average country.

**Supplementary Table 1. Results under various fixed effect specifications.** The top panel examines weight for age z-scores, while the bottom panel examines a binary variable for whether the child is underweight by WHO standards (below -2σ in weight for age). Standard errors are two-way clustered at the level of tropical year and admin1 (province) regions, and observations are reweighted using DHS sample weights and country size weights in order for estimates to represent an effect on the average country. Asterisks indicate statistical significance at the 1% (***), 5% (**) and 10% (*) levels (two-sided t-test, single hypothesis).

Supplementary Table 2. Results under various observation weighting options. 1-4 examine weight for age z-scores, while 5-8 examine a binary variable for whether the child is underweight by WHO standards (below -2σ in weight for age). Estimates are from OLS regressions with controls consisting of: fixed effects (indicators) for each country; country-specific controls for mother's age at child's birth and total years of mother's education; country-specific fixed effects for rural vs. urban; and UNICEF world region-specific linear trends in survey year as well as fixed effects for month of interview. Standard errors are two-way clustered at the level of tropical year and admin1 (province) regions. 1 and 5 use weights to represent the effect on an average country in the sample, 2 and 6 weigh observations to represent the average child in the sample; 3 and 7 use the same weights but exclude India given its dominance in the sample. 4 and 8 use no weights. Asterisks indicate statistical significance at the 1% (***), 5% (**) and 10% (*) levels (two-sided t-test, single hypothesis)

Supplementary Table 3. Results under various definitions of ENSO. 1-5 examine weight for age z-scores, while 6-10 examine a binary variable for whether the child is underweight by WHO standards (below -2σ in weight for age). (1) and (6) use the maximum monthly value observed over the full (May-April) tropical year. (2) and (7) use the maximum over the May-Dec tropical year; models (3) and (8) use the mean over the tropical year; and models (4) and (9) use the maximum of a three month rolling mean of monthly values observed during the full tropical year. (5) and (10) use indicator variables for El Niño-like and La Niña-like states designating all years where the maximum of a three month rolling mean of monthly values was greater than 0.5°C (Niño-like) or less than -0.5°C (Niña-like) from its reference climatology following NOAA CPC guidelines. Estimates are from OLS regressions with controls consisting of: fixed effects (indicators) for each country; country-specific controls for mother's age at child's birth and total years of mother's education; country-specific fixed effects for rural vs. urban; and UNICEF world region-specific linear trends in survey year as well as fixed effects for month of interview. Standard errors are two-way clustered at the level of tropical year and admin1 (province) regions, and observations are reweighted using DHS sample weights and country size weights in order for estimates to represent an effect on the average country. Asterisks indicate statistical significance at the 1% (***), 5% (**) and 10% (*) levels (two-sided t-test, single hypothesis).

Supplementary Table 4. Results across child age categories. Decomposing the effects of ENSO on weight-for-age (1), weight-for-height (2), and body mass index z-scores (3) shows a similar pattern across child age categories, despite smaller sample sizes leading to imprecise estimates. The same occurs for WHO threshold outcomes (4-5), including the likelihood of being underweight (below -2σ in weight for age) or wasted (below -2σ in weight for height). Estimates are from OLS regressions with controls consisting of: fixed effects (indicators) for each country; country-specific mother's age at child's birth, total years of mother's education, and rural vs. urban indicator; and UNICEF world region-specific linear trends in survey year and fixed effects for month of interview. Standard errors are two-way clustered at the level of tropical year and subnational administrative unit, and observations are reweighted using DHS sample weights and country size weights in order for estimates to be representative for an average country. Asterisks indicate statistical significance at the 1% (***), 5% (**) and 10% (*) levels (two-sided t-test, single hypothesis).

**Supplementary Table 5. Results as an average treatment effect.** Estimates are from OLS regressions with controls consisting of: fixed effects (indicators) for each country; country-specific controls for mother's age at child's birth and total years of mother's education; country-specific fixed effects for rural vs. urban; and UNICEF world region-specific linear trends in survey year as well as fixed effects for month of interview. Standard errors are two-way clustered at the level of tropical year and admin1 (province) regions, and observations are reweighted using DHS sample weights and country size weights in order for estimates to represent an effect on the average child in the sample countries. Asterisks indicate statistical significance at the 1% (***), 5% (**) and 10% (*) levels (two-sided t-test, single hypothesis).

**Supplementary Table 6. Results using decade fixed effects**.  Different anthropometric effects of ENSO are concentrated on short run measures (1-3) weight for age, weight for height, and body mass index z-scores, which all measure shorter-run effects of scarce nutrition, show evidence of contemporaneous ENSO effects measured in °C. Estimates are from OLS regressions with controls consisting of: fixed effects (indicators) for each country; country-specific controls for mother's age at child's birth and total years of mother's education; country-specific fixed effects for rural vs. urban; fixed effects for month of interview, and fixed effects for decade of survey year. Standard errors are two-way clustered at the level of tropical year and admin1 (province) regions, and observations are reweighted using DHS sample weights and country size weights in order for estimates to represent an effect on the average country. (4-5) WHO threshold outcomes show ENSO increases the likelihood of being underweight (below -2σ in weight for age) or wasted (below -2σ in weight for height).  Asterisks indicate statistical significance at the 1% (***), 5% (**) and 10% (*) levels (two-sided t-test, single hypothesis).

Supplementary Table 7. Results as a distributed lag model. Different anthropometric effects of ENSO are concentrated on short run measures (1-3) weight for age, weight for height, and body mass index z-scores, which all measure shorter-run effects of scarce nutrition, show evidence of contemporaneous ENSO effects measured in °C. Estimates are from OLS regressions with controls consisting of: fixed effects (indicators) for each country; country-specific controls for mother's age at child's birth and total years of mother's education; country-specific fixed effects for rural vs. urban; and UNICEF world region-specific linear trends in survey year as well as fixed effects for month of interview. Standard errors are two-way clustered at the level of tropical year and admin1 (province) regions, and observations are reweighted using DHS sample weights and country size weights in order for estimates to represent an effect on the average country. (4-5) WHO threshold outcomes show ENSO increases the likelihood of being underweight (below -2σ in weight for age), but shows a statistically insignificant effect on wasting (below -2σ in weight for height). Asterisks indicate statistical significance at the 1% (***), 5% (**) and 10% (*) levels (two-sided t-test, single hypothesis).

**Supplementary Table 8. Persistent stunting effects among older children**. For children between ages 2-5, height for age and likelihood of being below the WHO threshold for stunting (below -2σ in height for age) are impacted by ENSO state measured in °C during year of birth and during two following years. Estimates are from OLS regressions with controls consisting of: fixed effects (indicators) for each country; country-specific controls for mother's age at child's birth and total years of mother's education; country-specific fixed effects for rural vs. urban; and UNICEF world region-specific linear trends in survey year as well as fixed effects for month of interview. Standard errors are two-way clustered at the level of tropical year and admin1 (province) regions, and observations are reweighted using DHS sample weights and country size weights in order for estimates to represent an effect on the average country. Asterisks indicate statistical significance at the 1% (***), 5% (**) and 10% (*) levels (two-sided t-test, single hypothesis).

**Supplementary Table 9. Results with precipitation teleconnected countries added**, effects on (**1-3**) weight for age, weight for height, and body mass index z-scores, which all measure shorter-run effects of scarce nutrition, show evidence of contemporaneous ENSO effects measured in °C. Estimates are from OLS regressions with controls consisting of: fixed effects (indicators) for each country; country-specific controls for mother's age at child's birth and total years of mother's education; country-specific fixed effects for rural vs. urban; and UNICEF world region-specific linear trends in survey year as well as fixed effects for month of interview. Standard errors are two-way clustered at the level of tropical year and admin1 (province) regions, and observations are reweighted using DHS sample weights and country size weights in order for estimates to represent an effect on the average country. (4-5) WHO threshold outcomes show ENSO increases the likelihood of being underweight (below -2σ in weight for age) and wasted (below -2σ in weight for height).  Asterisks indicate statistical significance at the 1% (***), 5% (**) and 10% (*) levels (two-sided t-test, single hypothesis).

**Supplementary Table 10. Results including only locations teleconnected through both precipitation and temperature**, effects on (**1-3**) weight for age, weight for height, and body mass index z-scores, which all measure shorter-run effects of scarce nutrition, show evidence of contemporaneous ENSO effects measured in °C. Estimates are from OLS regressions with controls consisting of: fixed effects (indicators) for each country; country-specific controls for mother's age at child's birth and total years of mother's education; country-specific fixed effects for rural vs. urban; and UNICEF world region-specific linear trends in survey year as well as fixed effects for month of interview. Standard errors are two-way clustered at the level of tropical year and admin1 (province) regions, and observations are reweighted using DHS sample weights and country size weights in order for estimates to represent an effect on the average country. (4-5) WHO threshold outcomes show ENSO increases the likelihood of being underweight (below -2σ in weight for age) and wasted (below -2σ in weight for height).  Asterisks indicate statistical significance at the 1% (***), 5% (**) and 10% (*) levels (two-sided t-test, single hypothesis).

Supplementary Table 11. Countries and surveys in sample.


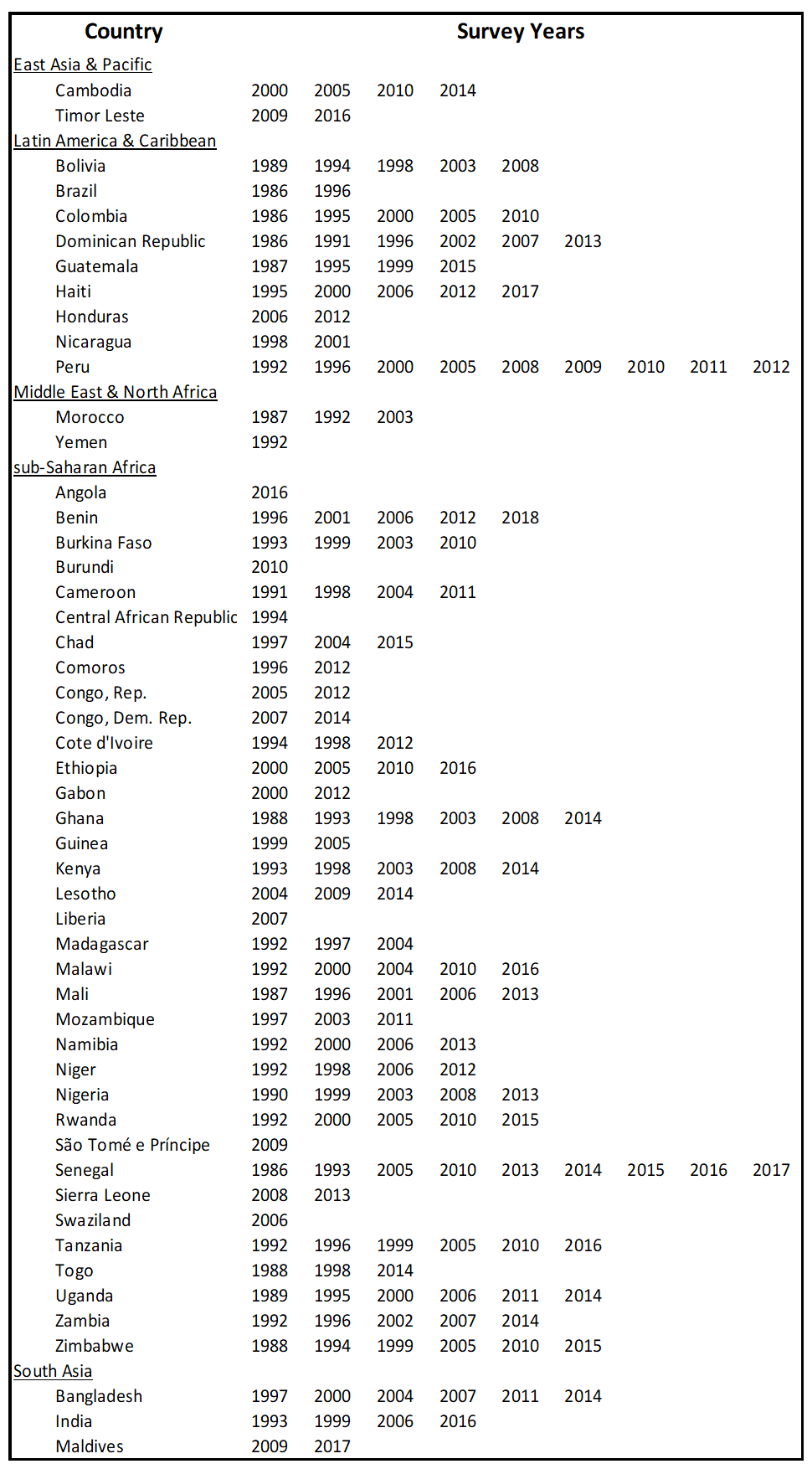


**Supplementary Table 12. Results adding country-month fixed effects**.  Weight-for-age (1), weight-for-height (2), and body mass index z-scores (3), which all measure shorter-run effects of scarce nutrition, show evidence of contemporaneous ENSO effects measured in °C. Estimates are from OLS regressions with controls consisting of: fixed effects (indicators) for each country; country-specific controls for mother's age at child's birth and total years of mother's education; country-specific fixed effects for rural vs. urban; country-specific fixed effects for month of interview, and linear and quadratic trends in survey year. Standard errors are two-way clustered at the level of tropical year and admin1 (province) regions, and observations are reweighted using DHS sample weights and country size weights in order for estimates to represent an effect on the average country. Asterisks indicate statistical significance at the 1% (***), 5% (**) and 10% (*) levels (two-sided t-test, single hypothesis).

**Supplementary Table 13. Month of interview not associated to NINO3.4 Anomaly.** Estimate is from OLS regressions with controls consisting of: fixed effects (indicators) for each country; country-specific controls for mother's age at child's birth and total years of mother's education; country-specific fixed effects for rural vs. urban; and UNICEF world region-specific linear trends in survey year. Standard errors are two-way clustered at the level of tropical year and admin1 (province) regions, and observations are reweighted using DHS sample weights and country size weights in order for estimates to represent an effect on the average country.

**Supplementary Table 14. Alternative standard error clustering strategies**. Weight for age shows evidence of contemporaneous ENSO effects measured in °C.  Estimates are from OLS regressions with controls consisting of: fixed effects (indicators) for each country; country-specific controls for mother's age at child's birth and total years of mother's education; country-specific fixed effects for rural vs. urban; fixed effects for month of interview; and UNICEF world region-specific linear trends in survey year. Inference remains consistent whether standard errors are not clustered (1); clustered at UNICEF region (2), country (3) or admin1/state (4) to adjust for arbitrarily serial and spatial autocorrelation at these geographic units, or two-way clustered at UNICEF region & decade (5), country and year (6), or admin1 and year (7, the main specification). Observations are reweighted using DHS sample weights and country size weights in order for estimates to represent an effect on the average country. Asterisks indicate statistical significance at the 1% (***), 5% (**) and 10% (*) levels (two-sided t-test, single hypothesis).

**Supplementary Table 15. Logistic regressions for dichotomous outcome variables.**  WHO threshold outcomes show a warmer ENSO state increases the likelihood of being underweight (below -2σ in weight for age), but shows a weaker, statistically insignificant effect on wasting (below -2σ in weight for height). Estimates (1) and (3) are from a linear probability model (LPM) using OLS with controls consisting of: fixed effects (indicators) for each country; country-specific mother's age at child's birth, total years of mother's education, and rural vs. urban indicator; and UNICEF world region-specific linear trends in survey year and fixed effects for month of interview. Standard errors are two-way clustered at the level of tropical year and subnational administrative unit, and observations are reweighted using DHS sample weights and country size weights in order for estimates to be representative for an average country. Asterisks indicate statistical significance at the 1% (***), 5% (**) and 10% (*) levels (two-sided t-test, single hypothesis). Columns (2) and (4) implement logistic regressions with the same fixed effect structure and observation weights, and standard errors clustered by first administrative unit-tropical year. The resulting odds ratios lead to the same qualitative interpretation as in the linear probability models of (1) and (3).

**Supplementary References**

1. Alderman H., Headey D. The timing of growth faltering has important implications for observational analyses of the underlying determinants of nutrition outcomes. *PLoS ONE* **13**, e0195904 (2018).
2. Cameron, A., Gelbach, J., Miller, D., Robust inference with multiway clustering. *Journal of Business & Economic Statistics* **29**, 238-249 (2011).
3. Hsiang, S.M., Jina, A.S., The Causal Effect of Environmental Catastrophe on Long-Run Economic Growth: Evidence From 6,700 Cyclones, *National Bureau of Economic Research Working Paper Series,* <http://www.nber.org/papers/w20352> (2014)
4. Bhutta, Z.A., Das, J.K., Rizvi, A., Gaffey, M.F., Walker, N., Horton, S., Webb, P., Lartey, A., Black, R.E., T.L.N.I.R. Group & Maternal and Child Nutrition Study Group. Evidence-based interventions for improvement of maternal and child nutrition: what can be done and at what cost? *The Lancet* **382**, 452-77 (2013).

1. UNICEF regions consist of Central and Eastern Europe and the Commonwealth of Independent States; East Asia and Pacific; Latin America and the Caribbean; Middle East and North Africa; Sub-Saharan Africa; and South Asia. [↑](#footnote-ref-2)
