# Supplementary material for: ENSO impacts child undernutrition in the global tropics
Source: Nat Commun. 2021 Oct 12;12:5785. doi: 10.1038/s41467-021-26048-7 (PMC8511020; doi:10.1038/s41467-021-26048-7)
Supplement: Supplementary file 3 — Reporting Summary [file 41467_2021_26048_MOESM3_ESM.pdf]

nature research

Corresponding author(s): Amir S. Jina

Last updated by author(s): Aug 20, 2021

Reporting Summary

Nature Research wishes to improve the reproducibility of the work that we publish. This form provides structure for consistency and transparency in reporting. For further information on Nature Research policies, see our Editorial Policies and the Editorial Policy Checklist.

Statistics

For all statistical analyses, confirm that the following items are present in the figure legend, table legend, main text, or Methods section.

n/a	Confirmed	
☐	☒	The exact sample size (*n*) for each experimental group/condition, given as a discrete number and unit of measurement
☐	☒	A statement on whether measurements were taken from distinct samples or whether the same sample was measured repeatedly
☐	☒	The statistical test(s) used AND whether they are one- or two-sided<br>*Only common tests should be described solely by name; describe more complex techniques in the Methods section.*
☐	☒	A description of all covariates tested
☐	☒	A description of any assumptions or corrections, such as tests of normality and adjustment for multiple comparisons
☐	☒	A full description of the statistical parameters including central tendency (e.g. means) or other basic estimates (e.g. regression coefficient) AND variation (e.g. standard deviation) or associated estimates of uncertainty (e.g. confidence intervals)
☐	☒	For null hypothesis testing, the test statistic (e.g. *F*, *t*, *r*) with confidence intervals, effect sizes, degrees of freedom and *P* value noted<br>*Give P values as exact values whenever suitable.*
☒	☐	For Bayesian analysis, information on the choice of priors and Markov chain Monte Carlo settings
☒	☐	For hierarchical and complex designs, identification of the appropriate level for tests and full reporting of outcomes
☐	☒	Estimates of effect sizes (e.g. Cohen's *d*, Pearson's *r*), indicating how they were calculated

Our web collection on statistics for biologists contains articles on many of the points above.

Software and code

Policy information about availability of computer code

Data collection	Data were downloaded and compiled using Stata 15 and 16.
Data analysis	Data were analyzed using Stata 16, QGIS 2.18, and Matlab 2018b.

For manuscripts utilizing custom algorithms or software that are central to the research but not yet described in published literature, software must be made available to editors and reviewers. We strongly encourage code deposition in a community repository (e.g. GitHub). See the Nature Research guidelines for submitting code & software for further information.

Data

Policy information about availability of data

All manuscripts must include a data availability statement. This statement should provide the following information, where applicable:

- Accession codes, unique identifiers, or web links for publicly available datasets
- A list of figures that have associated raw data
- A description of any restrictions on data availability

The raw survey data are subject to a user agreement and are available at available from the Demographics and Health Surveys Program at https://dhsprogram.com. The raw ENSO data are available via NOAA at https://www.cpc.ncep.noaa.gov/data/indices/. The processed ENSO data are available on Zenodo at https://doi.org/10.5281/zenodo.5208080. The University of Delaware gridded weather data are available at http://climate.geog.udel.edu/~climate/html_pages/archive.html. All other datasets produced or used in this analysis can be found at https://doi.org/10.5281/zenodo.5208080

Field-specific reporting

Please select the one below that is the best fit for your research. If you are not sure, read the appropriate sections before making your selection.

- [ ] Life sciences
- [x] Behavioural & social sciences
- [ ] Ecological, evolutionary & environmental sciences

For a reference copy of the document with all sections, see nature.com/documents/nr-reporting-summary-flat.pdf

Behavioural & social sciences study design

All studies must disclose on these points even when the disclosure is negative.

Study description	This study analyzes the relationship between the El Nino Souther Oscillation and child undernutrition. It is a quantitative, panel data analysis using existing and publicly accessible data.
Research sample	The research sample comprises children under the age of 5 years in households in 51 different countries. Data were collected in 186 separate household surveys and surveys are representative at the level of the first administrative division within a country. Children under age 5 are chosen because they have had weight and height measured due to their susceptibility to negative effects of nutritional deficiencies. Data are available from the Demographics and Health Surveys Program at https://dhsprogram.com/.
Sampling strategy	Details of the sampling procedure are available from the Demographics and Health Surveys (DHS) Program. Households have been randomly sampled within a country by DHS at intervals since the 1980s. Our main analysis yields a sample of 1,253,176 children. This sample is arrived at by compiling every survey in the DHS that met the following three criteria: 1) they fell in our range of years of analysis (1986-2018), 2) had age and weight measurements for under-5s in the data, and 3) fell within countries we identified as teleconnected with ENSO. No other criteria were used in selecting surveys from the DHS database.
Data collection	Survey data were collected by the Demographics and Health Surveys (DHS) Program. Survey instruments and methodology can be found here: https://dhsprogram.com/methodology/survey-types/DHS-Methodology.cfm. Data were collected for general purposes of tracking demographics and health among lower income populations, and no survey participant or enumerator was aware of the hypothesis of the current study. Climate data were collected by the National Oceanic and Atmospheric Administration (NOAA) and the University of Delaware (UDEL).
Timing	The earliest survey included in our data is from 1986. The latest is from 2018.
Data exclusions	No data for which weights and ages for children were recorded was dropped from the sample. This yields a sample of 1,253,176 children. For a secondary analysis, data on children with height and age or height and weight measurements are used. This yields samples of 1,218,846 and 1,205,335 respectively.
Non-participation	No participants dropped out/declined participation.
Randomization	Participants were not allocated to random groups.

Reporting for specific materials, systems and methods

We require information from authors about some types of materials, experimental systems and methods used in many studies. Here, indicate whether each material, system or method listed is relevant to your study. If you are not sure if a list item applies to your research, read the appropriate section before selecting a response.

Materials & experimental systems

n/a	Involved in the study	
x		Antibodies
x		Eukaryotic cell lines
x		Palaeontology and archaeology
x		Animals and other organisms
	x	Human research participants
x		Clinical data
x		Dual use research of concern

Methods

n/a	Involved in the study	
x		ChIP-seq
x		Flow cytometry
x		MRI-based neuroimaging

Human research participants

Policy information about studies involving human research participants

Population characteristics	See "Research sample" above.
Recruitment	See "Data collection" above.
Ethics oversight	Data were obtained from pre-existing survey dataset that were created between the years 1986 and 2018. Data are public use and de-identified, and as such do not require IRB review.

Note that full information on the approval of the study protocol must also be provided in the manuscript.
